# Conversion-oriented multimodal therapy enabling definitive surgery in a patient with FIGO stage IVA cervical cancer at high risk of fistula formation: a case report

**DOI:** 10.3389/fimmu.2026.1853610

**Published:** 2026-06-17

**Authors:** Yanling Yuan, Wanming He, Lihua Tong, Yu Yang, Wen Yang

**Affiliations:** 1 Department of Oncology, The Sixth Affiliated Hospital, School of Medicine, South China University of Technology, Foshan, Guangdong, China; 2 Department of Radiation Oncology, Dongguan Hospital of Traditional Chinese Medicine, Dongguan, Guangdong, China

**Keywords:** image-guided radiotherapy, immune checkpoint inhibitors, immunotherapy, operative, rectovaginal fistula, surgical procedures, uterine cervical neoplasms

## Abstract

**Background:**

FIGO stage IVA cervical cancer is frequently associated with extensive pelvic organ invasion and high treatment complexity. In selected patients with severe hemorrhage, renal dysfunction, or poor baseline condition, delivery of standard therapy may be clinically challenging.

**Case Presentation:**

We report a patient with FIGO stage IVA cervical squamous cell carcinoma presenting with massive vaginal bleeding, severe anemia, and renal dysfunction caused by extensive invasion of the rectum, bladder, distal ureter, and lower vagina. Emergent hemostatic radiotherapy achieved rapid bleeding control and clinical stabilization. Subsequently, an individualized conversion-oriented multimodal strategy integrating radiotherapy, targeted therapy, immunotherapy, and later consolidation chemotherapy was adopted with surgery as the intended endpoint. During treatment, serial image-guided radiotherapy (IGRT) monitoring prompted repeat CT simulation, target volume recontouring, and adaptive replanning according to tumor regression, enabling progressive reduction of irradiated target volumes while limiting unnecessary radiation exposure to adjacent pelvic organs. Marked tumor regression was achieved, followed by definitive radical surgery and pathologic complete response without recurrence during follow-up.

**Conclusion:**

This case suggests that, in highly selected patients with FIGO stage IVA cervical cancer, an individualized conversion-oriented multidisciplinary strategy incorporating IGRT-based monitoring and adaptive replanning may facilitate subsequent radical surgery and durable disease control. However, further clinical validation is required.

## 1 Introduction

Cervical cancer remains one of the most common malignant tumors among women globally, ranking fourth in incidence and fifth in mortality (1). Among FIGO IVA stage patients, extensive tumor invasion into adjacent organs such as the rectum and bladder often leads to severe local symptoms and complex complications, significantly increasing treatment difficulty (2). Although concurrent chemoradiotherapy with platinum-based agents combined with intracavitary brachytherapy has become the standard treatment regimen for locally advanced cervical cancer (3), its efficacy and safety remain challenging in IVA-stage patients. Particularly, the high risk of treatment-related fistula formation and long-term compromised quality of life persistently hinder the optimization of therapeutic strategies (4).

In recent years, immunotherapy has emerged as a promising therapeutic strategy for cervical cancer because of its virus-associated immunogenic characteristics. Immune checkpoint inhibitors targeting the PD-1/PD-L1 axis have demonstrated durable antitumor activity in recurrent, metastatic, and locally advanced cervical cancer, gradually reshaping systemic treatment paradigms (5–7). More recently, dual immune checkpoint blockade represented by cadonilimab has further expanded potential treatment options for advanced cervical cancer (8).

In clinical practice, some IVA-stage patients present with massive hemorrhage, renal impairment, or other critical organ dysfunction at initial diagnosis, making conventional platinum-based concurrent chemoradiotherapy or immediate radical surgery difficult to perform safely. Against this backdrop, treatment solely targeting local control often fails to achieve sustained benefits. The key challenge in managing these patients lies in stabilizing their condition while creating conditions for subsequent curative interventions. In recent years, with advances in radiotherapy techniques and the expansion of systemic treatment modalities, integrated therapeutic strategies targeting “conversion to radical surgery” have gained increasing attention (9, 10). However, clinical experience and reports remain limited in cervical cancer, particularly among IVA-stage patients at high risk of fistula formation (11).

Radiation therapy holds a central position in the comprehensive management of cervical cancer, serving not only for tumor control but also playing a vital role in scenarios such as emergency hemostasis. Concurrently, immunotherapy has demonstrated antitumor activity in recurrent or metastatic cervical cancer, providing a theoretical basis for exploring its application in earlier treatment stages (5, 6, 8). Epidermal growth factor receptor (EGFR) is highly expressed in approximately 90% of cervical squamous cell carcinomas, mediating tumor cell radioresistance and invasive metastasis (12). Nimotuzumab, a humanized monoclonal antibody, enhances radiotherapy sensitivity by blocking the EGFR signaling pathway (13). Studies have confirmed that its combination with chemoradiotherapy improves local control rates and progression-free survival (PFS) (14, 15). However, for patients with high tumor burden, complex anatomical invasion, and compromised organ function, there remains no established paradigm for safely and effectively integrating multiple therapeutic modalities while dynamically adjusting treatment strategies to reduce complication risks.

Against this backdrop, we report a case of FIGO IVA stage cervical squamous cell carcinoma with high risk of fistula formation. Following emergency hemorrhage control radiotherapy, the patient underwent individualized comprehensive treatment targeting conversion to radical surgery. Radiotherapy was re-planned multiple times based on imaging assessments during treatment, ultimately enabling successful completion of radical surgery and achieving pathological complete remission. This case study explores the therapeutic concept of conversion-oriented strategy in high-risk locally advanced cervical cancer and its potential implications for improving patient outcomes and quality of life.

## 2 Case report

A 59-year-old woman presented in Jan. 13, 2023 with a 1-week history of progressive fatigue and exertional dyspnea. Initial laboratory evaluation revealed severe anemia (hemoglobin 4.3 g/dL) and marked renal dysfunction (serum creatinine 5.87 mg/dL, blood urea nitrogen 44.4 mg/dL, uric acid 585 μmol/L). Further history disclosed a 6-month course of intermittent hematochezia and melena.

**Figure 1:**
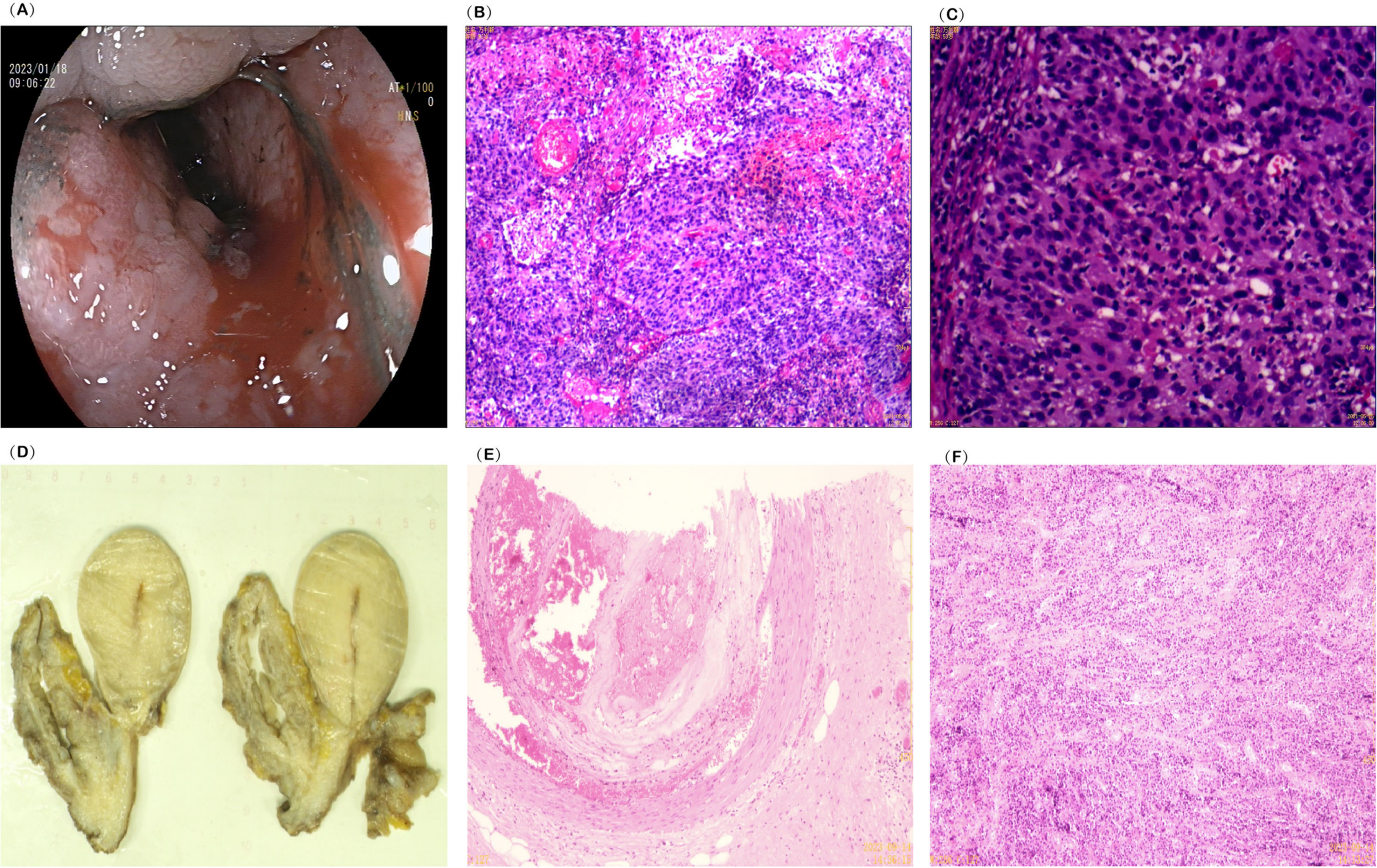
Endoscopic, histopathologic, and postoperative specimen findings. **(A)** Colonoscopic view showing a circumferential, friable rectal mass with mucosal congestion and surface bleeding, located approximately 10 cm from the anal verge. **(B, C)** Histopathologic examination of biopsy specimens demonstrating squamous cell carcinoma. **(D)** Gross postoperative specimen following radical surgery. **(E, F)** Postoperative histopathologic evaluation showing no residual viable tumor cells, consistent with a pathologic complete response (ypCR) (hematoxylin and eosin staining).

In view of gastrointestinal bleeding symptoms, further diagnostic evaluation was undertaken. Colonoscopy on Jan. 16, 2023 revealed a circumferential rectal mass approximately 10 cm from the anal verge, extending distally toward the anal canal, with a hyperemic, nodular, and friable appearance accompanied by spontaneous and contact bleeding; biopsy specimens were obtained (**Figure 1A**). Subsequent gynecologic ultrasonography demonstrated an enlarged uterus with heterogeneous echogenicity, suggestive of a pelvic space-occupying lesion. On Jan. 18, 2023, gynecologic examination identified extensive tumor involvement of the lower third of the vagina, a crater-like friable lesion at the vaginal apex with loss of identifiable cervical anatomy, a fixed pelvis, and a palpable rectal mass. Vaginal tumor biopsy was complicated by profuse hemorrhage, with an estimated blood loss of approximately 600 mL, necessitating emergency intervention.

**Figure 2:**
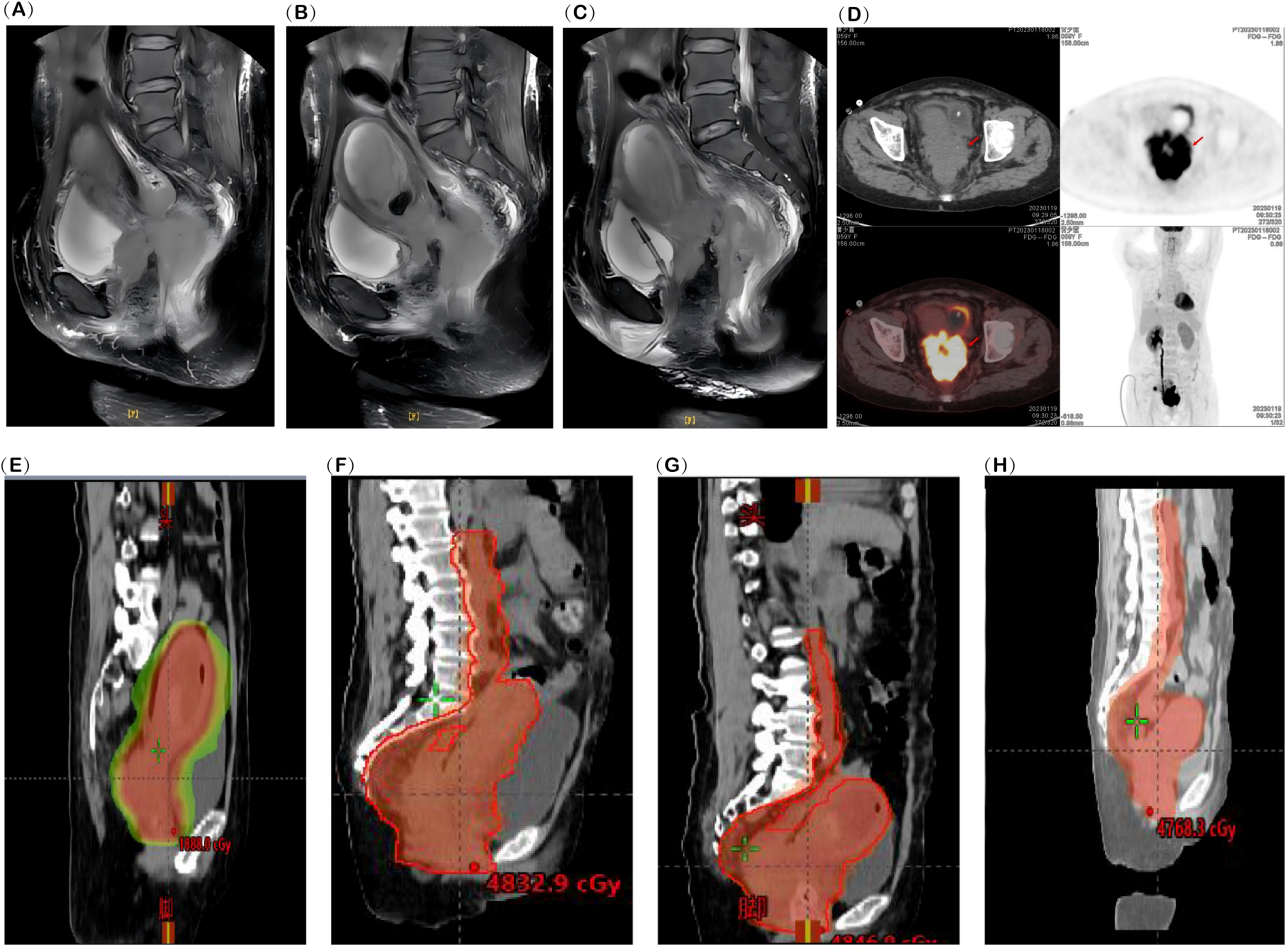
Baseline clinical presentation, multimodal imaging findings, emergency hemostatic radiotherapy, and adaptive radiotherapy planning during treatment. **(A)** Gynecological examination demonstrating a large exophytic cervical mass with active vaginal bleeding. **(B, C)** Baseline pelvic magnetic resonance imaging (MRI) demonstrating a bulky cervical tumor involving the uterine body and lower third of the vagina, with invasion of the rectum and posterior bladder wall. **(D)** Baseline positron emission tomography–computed tomography (PET–CT) showing intense metabolic activity in the primary cervical lesion with pelvic and para-aortic lymph node metastases, without evidence of distant organ metastasis. **(E)** Emergency hemostatic intensity-modulated radiotherapy (IMRT) plan delivered for rapid bleeding control. **(F)** Definitive pelvic external radiotherapy target delineation and dose distribution. **(G, H)** Adaptive replanning during treatment based on tumor regression and anatomical changes, demonstrating progressive reduction of irradiated target volume during serial treatment adaptation.

Given the uncontrolled bleeding and the patient’s compromised clinical status, emergent hemostatic radiotherapy was immediately initiated on Jan. 18, 2023 as a temporizing intervention for rapid bleeding control rather than definitive tumor treatment. Radiotherapy was delivered using a Varian VitalBeam linear accelerator (Varian Medical Systems, Palo Alto, CA, USA) with intensity-modulated radiotherapy (IMRT) and cone-beam computed tomography (CBCT)-based image guidance. IMRT was delivered to the primary cervical tumor and grossly involved adjacent tissues identified on imaging and clinical examination. The gross tumor volume (GTV) encompassed the primary cervical lesion and contiguous sites of gross invasion, including the uterus and upper-to-middle vagina. A 3-mm isotropic expansion from the GTV was applied to generate the planning target volume (PTV) to account for setup uncertainty. A total dose of 12 Gy in 2 fractions was delivered once daily over 2 consecutive days, with treatment completed on Jan. 19, 2023, resulting in rapid hemostasis and hemodynamic stabilization (**Figure 2E**). Because of severe anemia at presentation, the patient additionally required repeated packed red blood cell transfusions during the initial hospitalization period. Following hemostatic radiotherapy and supportive management, hemoglobin levels gradually stabilized, allowing subsequent multidisciplinary treatment planning and initiation of further oncologic therapy. Subsequent positron emission tomography–computed tomography (PET–CT), performed on Jan. 19, 2023, after the first fraction of emergent hemostatic radiotherapy, demonstrated a bulky cervical mass involving the uterine body and lower third of the vagina, with invasion of the rectum and posterior bladder wall, accompanied by pelvic and multiple para-aortic lymph node metastases, without evidence of distant organ metastasis (**Figure 2D**). Pelvic magnetic resonance imaging (MRI) further confirmed involvement of the bladder, rectum, and ureters (**Figures 2A–C**). Given the life-threatening hemorrhage and highly suspicious malignant imaging findings, emergency hemostatic radiotherapy was initiated before final histopathologic reporting became available. Subsequent histopathologic examination confirmed moderately differentiated cervical squamous cell carcinoma with rectal metastatic squamous cell carcinoma (**Figures 1A–C**). Based on the integrated clinical, radiologic, and pathologic findings, the patient was diagnosed with FIGO 2018 stage IVA cervical squamous cell carcinoma complicated by severe anemia, postrenal renal insufficiency, urinary tract infection, pulmonary infection, and hypoalbuminemia. Although distal ureteral obstruction-related renal dysfunction was present at admission, urine output remained preserved and no acute obstructive anuria requiring emergent decompression was observed. After multidisciplinary discussion, urinary diversion procedures were discussed with the patient and her family but were initially deferred because the patient expressed reservations regarding invasive drainage interventions during the early stabilization phase.

After clinical stabilization, definitive external radiotherapy was initiated on Jan. 20, 2023. The clinical target volume (CTV) included the cervical primary tumor region, pelvic lymphatic drainage areas, and para-aortic lymphatic drainage regions because of documented para-aortic lymph node metastases. A uniform 3-mm expansion from the CTV was applied to generate the PTV. The prescribed dose to the PTV was 45 Gy in 25 fractions (**Figure 2F**). During the radiotherapy phase, cadonilimab (250 mg every 3 weeks) and nimotuzumab (200 mg weekly) were administered concurrently according to the planned treatment schedule, with the aim of tumor downstaging and potential surgical conversion. Given the patient’s severe anemia, renal dysfunction, poor baseline condition, and ongoing hemorrhagic instability at presentation, cytotoxic chemotherapy was not incorporated during the initial treatment phase because of concerns regarding treatment tolerability during early clinical stabilization. A total of two cycles of cadonilimab were completed during radiotherapy. During administration of the third cycle on Mar. 7, 2023, an allergic reaction occurred, leading to treatment discontinuation. No additional cadonilimab was administered after radiotherapy discontinuation. To maintain continuity of systemic immunotherapy while improving treatment tolerability, the regimen was subsequently modified to adebrelimab, a PD-L1 inhibitor, combined with continued nimotuzumab. This treatment adjustment was primarily based on safety and tolerability considerations rather than an intentional shift in immunotherapeutic mechanism.

Cone-beam computed tomography (CBCT)-based image-guided radiotherapy (IGRT) was routinely performed approximately twice weekly throughout treatment to assess positioning accuracy, anatomical variation, and tumor regression. As substantial tumor shrinkage and anatomical changes became evident during treatment, repeat simulation CT scans were performed on Feb. 2 and Feb. 10, 2023, respectively, followed by adaptive replanning to accommodate progressive target volume reduction while maintaining adequate target coverage (**Figures 2G, H**).

During concurrent multimodal treatment, CTCAE v5.0 grade 4 hematologic toxicity developed after five fractions of radiotherapy, manifested as profound neutropenia (absolute neutrophil count <0.5 ×10^9^/L), necessitating intensive supportive care. The hematologic toxicity was considered multifactorial in the context of concurrent radiotherapy and systemic treatment. Radiation-induced proctitis subsequently emerged after ten fractions of treatment. Considering cumulative toxicity, anticipated impact on long-term quality of life, and the feasibility of subsequent surgical intervention, radiotherapy was electively discontinued after completion of 23 fractions. On Mar. 7, 2023, a rectovaginal fistula was identified, accompanied by progressive deterioration of pelvic organ function. The patient subsequently underwent bilateral ureteral stent placement and laparoscopic transverse colostomy on Mar. 14, 2023, to further stabilize urinary and gastrointestinal function and facilitate continuation of multidisciplinary treatment.

**Figure 3:**
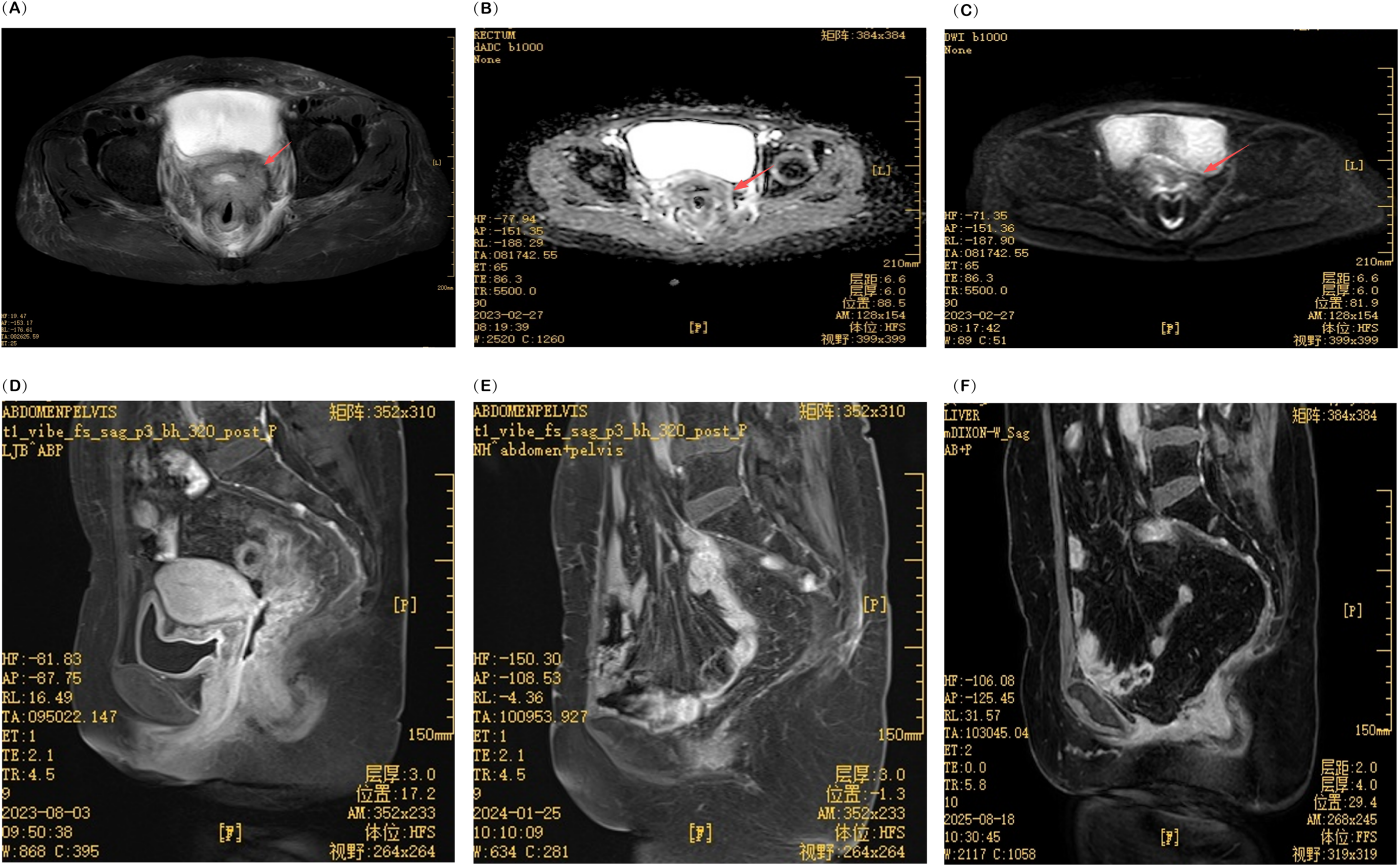
Serial MRI assessment of treatment response and follow-up. **(A–C)** Pelvic MRI performed on Feb. 27, 2023, after completion of radiotherapy, demonstrating marked tumor regression and substantial reduction of local invasion. **(D)** Follow-up pelvic MRI obtained on Aug. 3, 2023, showing no radiologic evidence of residual or recurrent disease. **(E)** Pelvic MRI on Jan. 5, 2024, confirming sustained complete response without signs of local recurrence. **(F)** Pelvic MRI on Aug. 18, 2025, demonstrating continued absence of tumor recurrence during long-term follow-up.

**Figure 4:**
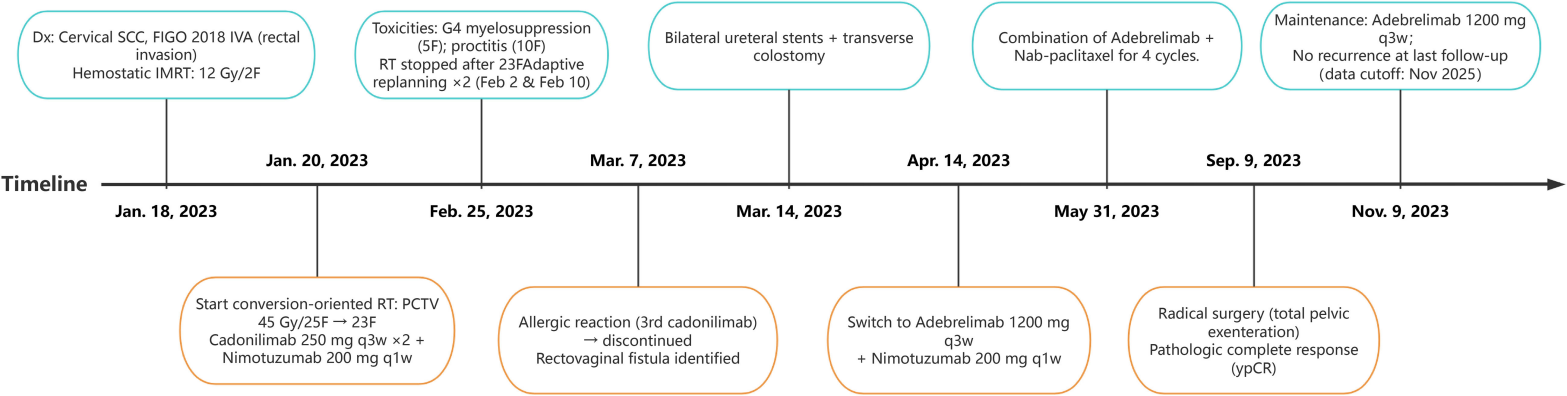
Timeline of treatment and clinical course. The patient was diagnosed with cervical squamous cell carcinoma, FIGO 2018 stage IVA, with rectal invasion on Jan. 18, 2023, and received emergent hemostatic intensity-modulated radiotherapy (IMRT; 12 Gy in 2 fractions) for uncontrolled vaginal bleeding. Conversion-oriented pelvic radiotherapy was initiated on Jan. 20, 2023 (planned PCTV 45 Gy in 25 fractions) in combination with cadonilimab and nimotuzumab. Treatment was discontinued after 23 fractions because of grade 4 myelosuppression and radiation proctitis; adaptive replanning was performed twice (Feb. 2 and Feb. 10, 2023). Cadonilimab was stopped after an allergic reaction, and a rectovaginal fistula was identified. The patient subsequently underwent bilateral ureteral stent placement and transverse colostomy on Mar. 14, 2023. Systemic therapy was switched to adebrelimab plus nimotuzumab on Apr. 14, 2023, followed by consolidation with nab-paclitaxel for four cycles starting May 31, 2023. Radical surgery (total pelvic exenteration) was successfully performed on Sep. 9, 2023, with postoperative pathology confirming a pathologic complete response (ypCR). Maintenance therapy with adebrelimab was continued thereafter, with no evidence of disease recurrence at the last follow-up (data cutoff: Nov. 2025).

The systemic treatment strategy was then modified, with initiation of adebrelimab combined with nimotuzumab on Apr. 14, 2023. Following improvement in renal function and stabilization of the patient’s general condition, nab-paclitaxel was added on May 31, 2023, as consolidation systemic therapy aimed at further enhancing tumor regression and maintaining disease control before planned radical surgery. Serial response evaluations using pelvic magnetic resonance imaging (MRI) and contrast-enhanced computed tomography (CT) were initiated after completion of radiotherapy and subsequently repeated during systemic treatment and preoperative reassessment. These serial imaging assessments demonstrated substantial tumor regression and marked reduction of local invasion. However, given the patient’s initial extensive invasion of the bladder, distal ureter, and rectum, as well as the development of a rectovaginal fistula during treatment, concerns remained regarding persistent microscopic disease and long-term pelvic complications despite the favorable radiologic response. In addition, after repeated multidisciplinary discussions and detailed communication regarding treatment goals, risks, postoperative functional impact, and alternative management strategies, the patient and her family strongly preferred definitive surgical treatment. Radical surgery was therefore considered the most feasible approach to achieve complete en bloc resection, address complex pelvic organ involvement and fistula-related complications, and pursue durable local disease control. On Sep. 9, 2023, the patient successfully underwent radical surgery, including total hysterectomy, partial resection of the sigmoid colon and rectum, total cystectomy with ileal neobladder reconstruction, colostomy reversal, and bilateral salpingo-oophorectomy. Postoperative pathology confirmed a pathologic complete response (ypCR) (**Figures 1D–F**). Maintenance therapy with adebrelimab was continued during follow-up as an individualized treatment decision based on sustained clinical benefit and acceptable tolerability through Nov. 2025, and follow-up to date has shown no evidence of tumor recurrence (**Figure 3**). The overall treatment timeline is summarized in **Figure 4**.

## 3 Discussion

Patients with FIGO stage IVA cervical cancer frequently exhibit extensive invasion of adjacent pelvic organs, including the bladder and rectum, resulting in highly complex therapeutic challenges. Concurrent chemoradiotherapy Concurrent chemoradiotherapy combined with brachytherapy remains the established standard treatment for locally advanced cervical cancer. However, in selected patients with extensive pelvic organ invasion, severe hemorrhage, renal dysfunction, poor baseline condition, or treatment-related complications during therapy, delivery of full standard treatment may become clinically challenging during multidisciplinary management.

This patient had FIGO stage IVA cervical squamous cell carcinoma and presented in critical condition, characterized by massive vaginal bleeding, significant renal dysfunction, and extensive tumor invasion of both the rectum and bladder. Although concurrent chemoradiotherapy combined with brachytherapy remains the standard treatment approach for locally advanced cervical cancer, delivery of full standard therapy in this individual patient was considered clinically challenging because of the patient’s unstable general condition, severe pelvic organ invasion, renal dysfunction, and subsequent treatment-related complications encountered during multidisciplinary management. In the present case, the therapeutic objective was not strict anatomical organ preservation, but rather maintenance of treatment feasibility, stabilization of pelvic organ function, and preservation of an opportunity for potentially curative intervention through a staged conversion-oriented strategy. Consequently, the central therapeutic challenge in this case was to stabilize the disease in the short term, achieve meaningful tumor downstaging in the intermediate phase, and ultimately create a window for definitive surgical intervention. In this context, the treatment strategy gradually evolved toward a conversion-oriented approach with surgery as the intended endpoint. In this report, the term “conversion-oriented” refers to an individualized multimodal treatment strategy aimed at achieving tumor downstaging and subsequent surgical feasibility in a patient initially considered unsuitable for definitive radical surgery. Importantly, fistula formation in this setting is likely multifactorial, potentially related to both aggressive tumor invasion and intensive local treatment. Therefore, treatment decisions in this case were continuously individualized through multidisciplinary reassessment of tumor response, treatment toxicity, pelvic organ involvement, surgical feasibility, and patient/family preferences. Major therapeutic modifications were made through repeated shared discussions with the patient and her family. Given the lack of established conversion-oriented strategies for cervical cancer, this case is presented as an exploratory multidisciplinary management approach in a highly selected clinical setting rather than a proposed alternative standard of care.

First, the patient underwent IMRT with a hypofractionated regimen of 12 Gy in 2 fractions for massive vaginal bleeding, which resulted in rapid and effective hemostasis. This clinical response is consistent with the prompt vascular effects of radiotherapy on tumor-associated vasculature (16–18). Following successful control of active bleeding, a multidisciplinary team reassessment recognized that the primary tumor exhibited extensive local invasion. Continued escalation toward definitive-dose pelvic radiotherapy and brachytherapy was considered potentially associated with increased risk of pelvic tissue injury and fistula-related complications in the setting of extensive bladder and rectal invasion. Once such severe complications occur, definitive management would inevitably require surgical intervention. Accordingly, if tumor burden could be sufficiently reduced and local infiltration alleviated, concurrent resection of the primary lesion at the time of fistula repair might allow eradication of potential sources of recurrence. On this basis, the therapeutic objective evolved from sole emphasis on local disease control to a conversion strategy with surgery as the intended endpoint, aiming to achieve more durable and comprehensive local control.

Given the patient’s severe renal insufficiency and compromised clinical condition at presentation, platinum-based chemotherapy was initially deferred because of concerns regarding treatment tolerance and potential further deterioration of renal function. An individualized multimodal strategy integrating radiotherapy, targeted therapy, and immunotherapy was therefore adopted as an exploratory approach aimed at achieving disease stabilization and tumor downstaging. Following subsequent improvement in renal function and overall condition, nab-paclitaxel was incorporated as consolidation systemic therapy. Previous studies have suggested that anti-EGFR monoclonal antibodies may enhance radiosensitivity in cervical cancer when administered concurrently with conventional chemoradiotherapy, providing a potential biological rationale for incorporating targeted therapy into multimodal treatment strategies in selected patients (15, 19). In parallel, immune checkpoint inhibitors have shown antitumor activity in recurrent or metastatic cervical cancer, with a subset of patients achieving objective responses and durable disease control (6). Moreover, emerging clinical evidence indicates that incorporating immunotherapy into systemic treatment strategies for cervical cancer is associated with acceptable efficacy and safety in broader patient populations, providing a rationale for its exploration at earlier stages of disease management (5, 20).

During the conversion-oriented treatment course, image-guided radiotherapy (IGRT) and adaptive replanning were applied as complementary but distinct components of treatment management. Cone-beam computed tomography (CBCT)-based IGRT was routinely performed approximately twice weekly throughout radiotherapy to assess patient positioning accuracy, anatomical variation, and tumor regression during treatment. As substantial tumor regression and anatomical changes became evident on serial imaging assessments, repeat simulation CT scans were performed on Feb. 2 and Feb. 10, 2023, respectively, followed by target recontouring and adaptive replanning by the attending radiation oncologists and medical physicists. The primary purpose of replanning was to accommodate tumor shrinkage and progressive reduction of irradiated pelvic volume while maintaining adequate target coverage during treatment adaptation. Given the substantial initial tumor burden and the overarching therapeutic objective—not only to achieve tumor regression but also to preserve conditions favorable for subsequent radical surgery and fistula repair—radiotherapy was dynamically refined throughout treatment. This response-guided adaptive strategy facilitated continued tumor regression while helping preserve more favorable tissue conditions for subsequent surgical intervention (21).

Importantly, radiotherapy in the present case was not intended to replace standard definitive chemoradiotherapy for FIGO stage IVA cervical cancer, which conventionally includes full-dose pelvic irradiation combined with brachytherapy boost. Instead, radiotherapy was incorporated as part of a staged conversion-oriented strategy with radical surgery as the planned therapeutic endpoint. Given the patient’s extensive invasion of the rectum, bladder, distal ureter, and lower vagina, together with the subsequent development of a rectovaginal fistula during treatment, further escalation to definitive-dose chemoradiotherapy and brachytherapy was considered likely to increase pelvic tissue injury and compromise subsequent surgical feasibility. Therefore, after initial emergency hemostatic irradiation and subsequent pelvic external radiotherapy, brachytherapy was not pursued because of concerns regarding fistula aggravation, cumulative pelvic toxicity, and the already established plan for radical surgical resection.

Notably, the integration of radiotherapy and immunotherapy may provide an important biological basis for multimodal conversion-oriented treatment in selected patients with advanced cervical cancer. Previous studies have demonstrated that combining radiotherapy with immune checkpoint inhibitors—including anti–cytotoxic T-lymphocyte–associated protein 4 (anti–CTLA-4) and anti–programmed cell death protein 1 (anti–PD-1) agents—can elicit more robust antitumor responses than either modality alone, suggesting the presence of complementary immunoregulatory mechanisms among these treatments (22). Mechanistically, CTLA-4 blockade reduces immunosuppressive regulatory T-cell populations, while PD-1 inhibition alleviates functional exhaustion of CD8^+^ T cells. Concurrently, radiotherapy promotes the release of tumor-associated antigens and enhances T-cell receptor diversity within tumor-infiltrating lymphocytes, thereby amplifying antitumor immune responses (23).

Taking into account the patient’s substantial tumor burden, extensive anatomical invasion, and impaired renal function, a strategy integrating radiotherapy with targeted therapy and immunotherapy was implemented following emergent hemostatic irradiation. This approach not only reinforced local radiosensitization but also provided sustained immunologic support for systemic tumor control and continued tumor regression, ultimately enabling the achievement of conversion-oriented strategy.

Overall, this patient was successfully converted from an initially unresectable state to definitive radical surgery through an individualized, staged multimodal treatment strategy, ultimately achieving a pathologic complete response. This case suggests that, in selected patients with FIGO stage IVA cervical cancer who are unsuitable for standard treatment approaches because of extensive local invasion or compromised clinical condition, a conversion-oriented multimodal strategy may provide a potential opportunity for definitive surgery and durable disease control. However, the broader applicability of this approach remains uncertain and requires further clinical investigation.

This study represents a single-case report, and its conclusions are primarily derived from individualized clinical decision-making; therefore, the generalizability of the findings is inherently limited. The patient underwent a complex, staged, and sequential multimodal treatment course—including hemostatic radiotherapy, targeted therapy, immunotherapy, response-adaptive radiotherapy replanning, and subsequent surgical intervention—making it difficult to disentangle the independent contribution of each component to the overall success of conversion. In addition, this conversion-oriented strategy relies on advanced imaging assessment, sophisticated radiotherapy techniques, and close multidisciplinary collaboration. As such, its reproducibility and broader applicability across different clinical settings remain to be established and warrant further investigation.

## 4 Conclusion

For selected patients with FIGO stage IVA cervical cancer and extensive pelvic organ invasion, standard treatment delivery may become clinically challenging because of compromised general condition and complex local disease involvement. This case illustrates that an individualized, conversion-oriented multimodal strategy incorporating staged disease control, adaptive radiotherapy planning, and multidisciplinary reassessment may facilitate subsequent radical surgery in highly selected patients initially considered unsuitable for definitive surgical intervention. Such an exploratory approach may provide a potential opportunity for durable disease control in carefully selected clinical scenarios; however, its broader applicability remains uncertain and requires further clinical investigation.

## Data Availability

The original contributions presented in the study are included in the article/**Supplementary Material**. Further inquiries can be directed to the corresponding authors.
